# Hepcidin-Ferroportin Interaction Controls Systemic Iron Homeostasis

**DOI:** 10.3390/ijms22126493

**Published:** 2021-06-17

**Authors:** Elizabeta Nemeth, Tomas Ganz

**Affiliations:** 1 Department of Medicine, University of California, Los Angeles, CA 90095, USA; enemeth@mednet.ucla.edu; 2 Departments of Medicine and Pathology, University of California, Los Angeles, CA 90095, USA

**Keywords:** iron deficiency, iron overload, hemochromatosis, anemia, metal transport

## Abstract

Despite its abundance in the environment, iron is poorly bioavailable and subject to strict conservation and internal recycling by most organisms. In vertebrates, the stability of iron concentration in plasma and extracellular fluid, and the total body iron content are maintained by the interaction of the iron-regulatory peptide hormone hepcidin with its receptor and cellular iron exporter ferroportin (SLC40a1). Ferroportin exports iron from duodenal enterocytes that absorb dietary iron, from iron-recycling macrophages in the spleen and the liver, and from iron-storing hepatocytes. Hepcidin blocks iron export through ferroportin, causing hypoferremia. During iron deficiency or after hemorrhage, hepcidin decreases to allow iron delivery to plasma through ferroportin, thus promoting compensatory erythropoiesis. As a host defense mediator, hepcidin increases in response to infection and inflammation, blocking iron delivery through ferroportin to blood plasma, thus limiting iron availability to invading microbes. Genetic diseases that decrease hepcidin synthesis or disrupt hepcidin binding to ferroportin cause the iron overload disorder hereditary hemochromatosis. The opposite phenotype, iron restriction or iron deficiency, can result from genetic or inflammatory overproduction of hepcidin.

## 1. The Roles of Iron

Iron is an essential trace element for nearly all living organisms. Even though iron is one of the most abundant elements in the Earth’s crust, ferric iron, an oxidized form common in the oxygen-rich environment on the surface of the Earth, is poorly soluble and difficult to access by most life forms. Accordingly, biological organisms have evolved mechanisms that conserve iron and recycle it internally. In adult humans, total body iron content is approximately 3–4 g, whereas normal daily losses are only 1–2 mg. To remain in iron balance, healthy humans must absorb a similar amount of iron from their diets. 

The ability of iron to donate or accept an electron in cellular and extracellular environments is what makes it a versatile catalytic component of many enzymes involved in energy-producing bioreactions and critical biosynthetic pathways, as well as in enzymes that generate reactive oxygen species for host defense. Iron also coordinates oxygen in hemoglobin and myoglobin, molecules involved in oxygen transport and its cellular storage. In biological systems, iron carries out its function in association with three common types of moieties: iron coordinated by protein side chains, iron complexed within the porphyrin ring of heme, and iron within iron-sulfur clusters. Outside of these controlled chemical environments, iron displays promiscuous reactivity that can damage cells and tissues.

## 2. Iron Compartments and Flows

Biological organisms closely regulate intracellular and extracellular iron concentrations, navigating between the twin threats of inadequate iron supply that would limit critical functions, and uncontrolled iron excess that could be toxic to the organism. Systemic iron homeostasis, best understood in humans and laboratory rodents, is maintained by regulating intestinal iron absorption, the concentration of iron in blood plasma and extracellular fluid, the distribution of iron among organs and tissues, and the amount of iron kept in stores. Iron dyshomeostasis can manifest as total body iron deficit (iron deficiency) or excess (iron overload), as well as iron maldistribution among tissues in which individual tissues or organs may become iron-deficient or iron-overloaded. Such iron disorders may be caused by genetic lesions that directly impair iron regulation, or conditions that impact iron regulation indirectly. 

**Figure 1:**
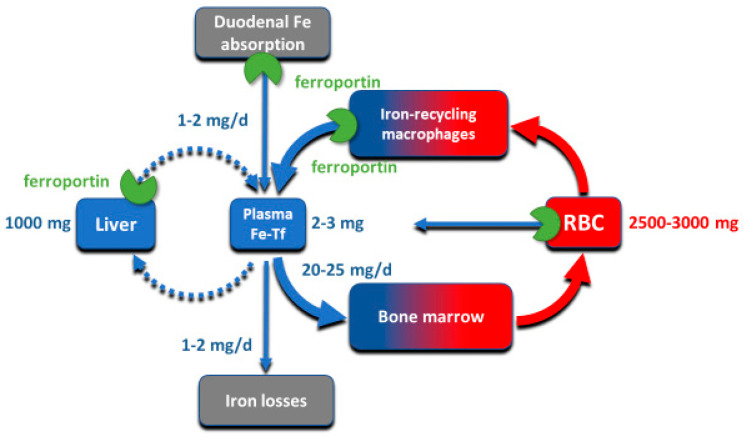
The key iron flows and compartments.

Although all cells in a multicellular organism contain iron, systemic iron homeostasis is primarily affected by the following compartments: the erythron (red blood cells and their precursors in erythropoietic organs), two types of stores (hepatocytes of the liver and macrophages of the spleen and the liver), blood plasma which moves iron between tissues and organs, and absorptive enterocytes in the duodenum through which iron enters the body, ordinarily to replace small losses from the body caused by shedding of iron-containing cells (Figure 1).

The largest of these iron compartments is the erythron (erythrocytes and their precursors), which contains approximately 2–3 g of iron, representing about 2/3–3/4 of the total body iron in adult humans. Erythroid iron is almost entirely contained within hemoglobin, at the concentration of 1 g of iron per liter of packed erythrocytes. The second largest compartment are the hepatocyte stores, where up to 1 g of iron is contained in cytoplasmic ferritin of hepatocytes. The stores are highly variable and can be nearly depleted in many women of reproductive age because of menstrual blood loss combined with low dietary intake. The recycling macrophages in the spleen, liver and marrow function as a rapid turnover compartment [1]. The spleen, which represents a substantial portion of the macrophage storage compartment, normally contains only about 0.05 g of iron but has the capacity for much larger amounts [2]. Only about 0.3 g of iron are contained in the other tissues, in myoglobin of muscles and in iron-containing enzymes.

Iron is transported around the body on blood plasma carrier protein transferrin, whose two iron-binding sites are normally 20–45% occupied. Each cell in the body has transferrin receptors (TfR1) that are endocytosed into acidified endosomes where iron dissociates from the TfR1-transferrin complex and is transported across the endosomal membrane into the cytoplasm. The transferrin compartment in blood plasma holds only 2–3 mg of iron but delivers to target tissues 20–25 mg/day so its iron content turns over every few hours. Despite changes in dietary iron content and tissue demand for iron, the concentration of iron in the blood plasma in healthy humans is remarkably stable, as is the total body iron content. Moreover, duodenal iron absorption increases several-fold after blood loss or exposure to hypoxia, or in response to iron deficiency. These observations provided early evidence that the absorption and tissue distribution of iron must be subject to endocrine regulation [3].

## 3. Molecular Basis of Systemic Iron Homeostasis

**Figure 2:**
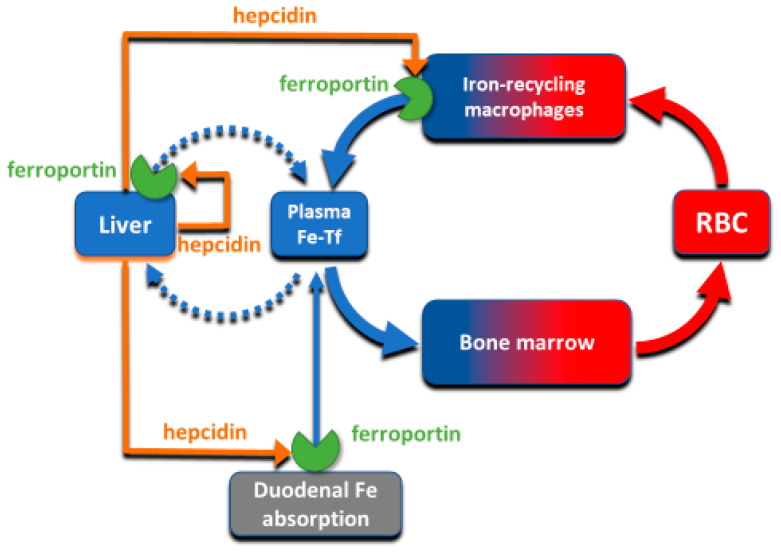
The interaction of hepcidin with ferroportin controls iron flows into plasma.

The absorption and tissue distribution of iron is principally controlled by the interaction of the hepatic hormone hepcidin with ferroportin. Ferroportin is expressed in iron-storing and iron-transporting tissues [4] and functions both as the hepcidin receptor and the sole known cellular exporter of elemental iron in multicellular organisms (Figure 2). The 25 amino-acid hepcidin peptide (MW 2.8 kD) is synthesized by hepatocytes and secreted into blood plasma, with concentrations in healthy humans ranging from approximately 2–20 nM [5,6], around hundred-fold higher than the concentration of the similarly-sized peptide hormones insulin, glucagon or parathyroid hormone. 

## 4. Ferroportin Structure 

**Figure 3:**
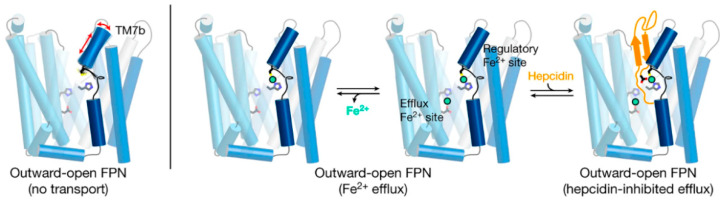
A current model of hepcidin (orange) interaction with ferroportin (blue) in which binding is dependent on iron (green). The framework of helices that make up ferroportin are depicted as cylinders connected by extracellular and intracellular disordered loops. The side chains that make up the binding sites for iron are shown. Modified from [14].

Ferroportin, a member of the solute carrier family, is systematically named SLC40A1. The human protein has 571 amino acids for a molecular weight of around 65–70 kD [7,8,9]. The variation in molecular mass is likely caused by tissue-specific glycosylation but the functional consequences of the glycosylation differences between the forms purified from the duodenal enterocytes, hepatocytes and macrophages are not yet understood [10]. Structurally, ferroportin consists of two 6-transmembrane-helix bundles (N-lobe and C-lobe) joined by a cytoplasmic loop, with both C- and N-termini located in the cytoplasm (Figure 3). The two helix bundles enclose a cavity through which iron is thought to exit the cell. The shape and similarity to more completely characterized family members suggest that ferroportin exports cellular iron by an alternating access mechanism, wherein ferroportin alternates between an open-inward conformation which binds intracellular iron, and an open-outward conformation which releases the iron to the extracellular space (Figure 3). The remarkable feat of structural characterization of a ferroportin from the bacterium *Bdellovibrio bacteriovorus* in both open-outward and open-inward conformations [11] represents strong support for such a model. The exported species is almost certainly ferrous iron [12] but the energetics of iron export is not yet well characterized. Because iron is exported against the force exerted by the transmembrane electric field, and there is no evidence of direct coupling of the transport to ATP hydrolysis, the export of iron is likely facilitated by the coupled “downhill” transport of another ion or small molecule [13].

Structural analyses of ferroportin indicate that there are two divalent metal-binding sites, one in each lobe, facing the internal cavity of ferroportin (Figure 3) [11,13,14]. How these sites mediate the export of iron is not yet clear. The site in the N-lobe can bind calcium which may have a modulatory role on iron transport [13]. Although calcium itself is not transported, it is required for human ferroportin transport activity, and is thought to directly bind to ferroportin and facilitate a conformational change critical to the transport cycle. Another area of interest is how cytoplasmic iron destined for export reaches ferroportin. Current evidence indicates that cytoplasmic iron is predominantly present as a complex of ferrous iron with reduced glutathione (summarized in [15]), and that the complex of ferrous iron and reduced glutathione may be delivered to ferroportin via the cytoplasmic iron chaperone PCBP2 [16]. 

## 5. Ferroportin Interactions with Hepcidin

The flow of iron out of the cells is controlled by hepcidin through two known mechanisms: occlusion of the open-outward conformation of ferroportin by hepcidin [17], and hepcidin-induced endocytosis and degradation of ferroportin [4]. The occlusion mechanism would be effective at hepcidin concentrations where most ferroportin molecules remain occluded by hepcidin most of the time, and would be rapidly reversible when hepcidin concentrations are decreased. The endocytosis mechanism could be initiated by even transient binding of hepcidin to ferroportin, and would cause the permanent removal of ferroportin from the cell surface that would require resynthesis of ferroportin for the recovery of iron transport. The second mechanism would therefore be expected to occur at lower concentrations of hepcidin, and to have a prolonged effect, even if hepcidin concentrations subsequently decrease. Although hepcidin peptide injected into mice is cleared from blood circulation within minutes, its plasma iron lowering effect lasts for 24–48 h, supporting the importance of the endocytic mechanism for ferroportin physiology [18].

Hepcidin regulation of ferroportin by the endocytic mechanism resembles the generic ligand-induced receptor endocytosis. It appears to require hepcidin-induced conformational change in ferroportin that triggers the ubiquitination of the lysine-rich cytoplasmic segment connecting the two 6-helix domains of ferroportin [19,20]. Ubiquitinated ferroportin is then targeted to lysosomes and proteasomes for degradation. A recent study [21] provide convincing evidence that *Rnf217* is an important E3 ubiquitin ligase that triggers the degradation of ferroportin in response to hepcidin binding.

Recent advances in structural understanding of the hepcidin-ferroportin interaction confirmed the simple models generated by targeted mutagenesis of hepcidin and ferroportin but also provided surprising new details [14]. In a nanodisc membrane model, hepcidin is seen to bind to the C-lobe of ferroportin (Figure 3), with the highly variable hepcidin loop largely extracellular, and the relatively conserved C- and N-termini of hepcidin deeply buried in the central cavity. The model also revealed an important unexpected feature, the indication that the suspected hepcidin-binding site in the C-lobe, centered on the critically important C325 thiol cysteine, utilizes an iron atom to coordinate hepcidin binding [14]. It is not yet clear how this contributes to hepcidin physiology but it can be anticipated that the mechanism could provide selectivity for hepcidin to bind to ferroportin molecules actively transporting iron as opposed to those that may be in a resting state. One indication that this mechanism may have a physiological effect is the recently reported inhibition of hepcidin-induced ferroportin degradation by the intracellular iron chelator deferiprone but not by the extracellularly acting chelator deferoxamine [22]. How the new structural model accommodates other hepcidin agonists ranging from minihepcidin peptides [23] to small molecules [24] remains to be determined.

## 6. Hepcidin Synthesis and Elimination

**Figure 4:**
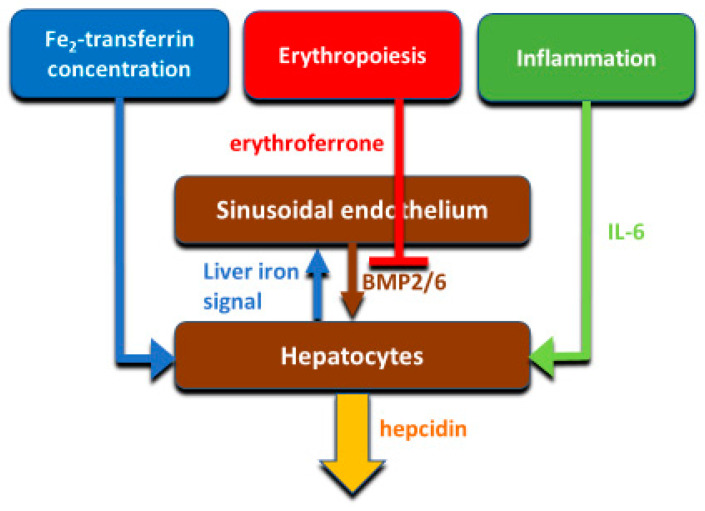
Iron, erythropoiesis and inflammation regulate hepcidin transcription through their effects on hepatocytes and by modulating the paracrine signaling between hepatic sinusoidal endothelium and hepatocytes.

Hepcidin production is principally transcriptionally regulated. The main systemic regulators of hepcidin include plasma iron concentrations (mainly through the interaction of diferric transferrin with transferrin receptors TFR1 and TFR2 in the liver), hepatic iron stores, systemic inflammation mainly communicated to hepatocytes by IL-6, and erythroid activity conveyed by the concentrations of the erythroid hormone erythroferrone (Figure 4). The mechanisms were reviewed in recent publications [25,26] and will not be discussed here.

The size of hepcidin and its mechanism of action suggest two catabolic mechanisms. One is efficient filtration of hepcidin as a small peptide through the renal glomerular membrane [27], followed by reuptake and degradation of filtered hepcidin in the proximal tubule, utilizing a generic mechanism for recycling the amino acid content of filtered proteins. Efficient filtration of hepcidin in the glomerulus is facilitated by the relatively low fraction of hepcidin that is bound by plasma protein, estimated at about 40% [28]. A small percentage of filtered hepcidin survives the uptake and degradation in the proximal tubule and can be detected in urine, similarly to other peptide hormones. In support of the important role of the kidneys in hepcidin removal, serum hepcidin concentrations are very high in patients with chronic renal failure, but are effectively lowered by hemodialysis [29]. This effect of hemodialysis is brief, as hepatic hepcidin synthesis is sufficiently rapid to restore hepcidin concentrations within hours. The second mechanism of hepcidin clearance depends on the endocytosis of the hepcidin-ferroportin complexes by the cells that express high concentrations of ferroportin [30,31]. The quantitative contribution of the two mechanisms as well as any possible alternative mechanisms of hepcidin catabolism are not known. 

## 7. Evidence for Exclusivity of the Hepcidin-Ferroportin Ligand-Receptor Dyad

**Figure 5:**
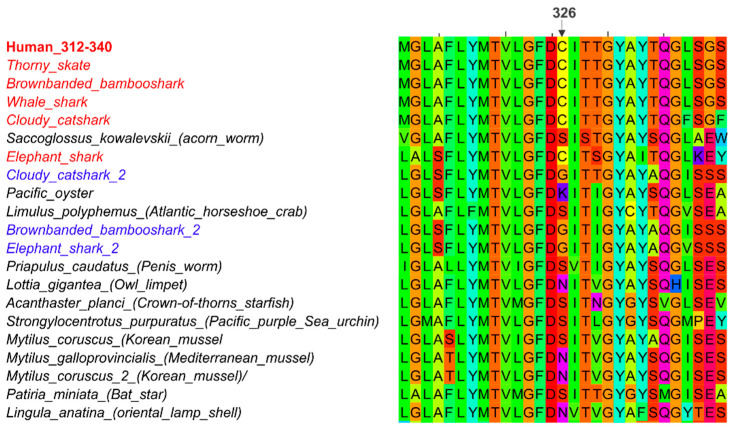
Although ferroportin is evolutionarily ancient, the thiol cysteine required for hepcidin binding by ferroportin appears first in early vertebrate ferroportins. Cartilaginous fish ferroportin that contains the C326-equivalent is denoted with species name in red. Second ferroportin in cartilaginous fish that has another amino acid in this position is denoted with species name in blue. Invertebrate ferroportin lacks C326 and is denoted with species name in black.

Ferroportin is evolutionarily ancient and can be found in plants and multicellular animals as simple as hydra. By contrast, hepcidin is a vertebrate hormone, with no known antecedent before fish. Remarkably, the essential conserved amino acid at the ferroportin binding site for hepcidin, C326 (human numbering), first appears in cartilaginous fish (Figure 5) some of which have a second ferroportin gene that has another amino acid in this position. C326 is strictly conserved in all vertebrates that have hepcidin but another amino acid is in this position in invertebrates, indicating that hepcidin and its binding site on ferroportin co-evolved. A second hepcidin gene is found in some vertebrates, mostly fish, but is not involved in iron regulation, and is hypothesized to have another function, perhaps as an antimicrobial peptide [32].

Consistent with the dyadic relationship between hepcidin and ferroportin, the isosteric C326S mutation in mice and humans, which causes complete loss of binding of hepcidin to ferroportin, phenocopies severe hepcidin deficiency [33]. It is therefore likely that hepcidin has no other nonredundant function than to bind to ferroportin and regulate its ability to transport iron. 

## 8. Regulation of Ferroportin by Intracellular Signals

In addition to systemic control of ferroportin by circulating hepcidin, ferroportin is also regulated by intracellular conditions. These local regulatory mechanisms may decrease toxicity from excessive cellular iron, preserve cellular iron under conditions of systemic iron deficiency, or amplify the effect of systemic hepcidin changes. In addition, inflammatory stimuli in the form of Toll-like receptor ligands can directly suppress cellular ferroportin mRNA levels and decrease cellular iron export to blood plasma [34]. Table 1 summarizes the local regulators of ferroportin and the setting in which their activities have been characterized. 

The IRE-IRP system functions in most cells to coordinate ferroportin translation with intracellular iron levels. Ferroportin mRNA contains 5′ IRE. Under conditions of iron deficiency, binding of IRP1/2 to 5′ IRE inhibits ferroportin translation, thus limiting iron export and conserving cellular iron. In duodenal enterocytes, however, the existence of ferroportin transcripts that lack the 5′ IRE would make these short-lived cells less responsive to their own cellular iron levels, and by implication more responsive to systemic iron requirements communicated to enterocytes by the concentration of hepcidin in blood plasma [35]. Low hepcidin levels during iron deficiency and anemia allow continuous iron export from enterocytes, resulting in activation of the enterocytes HIF system (specifically HIF2α), which further increases ferroportin transcription [38]. HIF2α also mediates increase in other mRNAs encoding proteins involved in the apical absorption of dietary iron (DCYTB and DMT1) thus leading to a coordinated increase in apical and basolateral iron transport and overall increase in duodenal iron absorption.

The 5′ IRE-lacking form of ferroportin mRNA is also found in erythroblasts, and the erythroid cells supply essential iron to other organs during iron deficiency [35]. Here ferroportin may also function to mitigate iron toxicity by releasing excess iron not used for hemoglobin synthesis. 

Iron-recycling macrophages, found in the spleen (but also in the liver and the marrow), are intermittently confronted with large boluses of hemoglobin that must be digested to release heme, which is then degraded via heme oxygenase to release iron. This process is dependent on the derepression of multiple genes involved in this process, including ferroportin that is required to export the recycled iron. The primary stimulus for the derepression of these genes is heme, sensed by the transcriptional repressor Bach1 (Btb And Cnc Homology 1) that binds heme and dissociates from its partner the small Maf protein bound to the promoter element MARE/ARE (Maf-recognition element/antioxidant response element). Bach1 is replaced by the transcriptional inducer Nrf2 which is stabilized by heme, translocated into the nucleus, and stimulates the transcription of the ferroportin gene and other genes involved in the recycling of heme to iron [36,41]. At high concentrations of iron, ferroportin mRNA and protein are increased, presumably because of oxidative stress causing Nrf2-mediated induction of ferroportin [42,43]. 

There is evidence that microRNAs such as miR-485-3p, miR-20a and miR-20b, can posttranscriptionally downregulate ferroportin but the physiological role of this mechanism is uncertain [37,39,40].

## 9. Local Functions of Ferroportin and Its Autocrine/Paracrine Regulation by Hepcidin

The “professional” iron exporting cell types, i.e., duodenal enterocytes, iron-recycling macrophages and iron-storing hepatocytes, all deliver iron to blood plasma for use in hemoglobin synthesis by erythroblasts as well as for other uses in iron-consuming tissues. However, ferroportin is also found in other tissues where it appears to serve a cell-autonomous function of releasing unneeded and potentially toxic iron. 

Ferroportin is expressed at a remarkably high level in erythroblasts and mature erythrocytes. In erythroblasts, it may release unobligated iron and here it is subject to hepcidin regulation [44]. In mature erythrocytes, which lack an endocytic mechanism and may therefore be less sensitive to inhibition of iron export by systemic hepcidin, ferroportin may serve as a safety valve to release labile iron leaking from hemoglobin molecules damaged by repeated cycling between high and low oxygen states. The importance of these mechanism is illustrated by the adverse consequences of erythroid-specific disruption of the ferroportin gene in mice, which suffer from mild hemolytic anemia caused by increased sensitivity of erythrocytes to hemolytic stress [45,46].

Ferroportin is required for normal functioning of cardiac myocytes [47], presumably to remove excess iron. Cardiac myocytes also produce hepcidin but this does not contribute significantly to systemic hepcidin levels. Rather, hepcidin acts as an autocrine or paracrine regulator of ferroportin that may protect tissues from loss of iron through ferroportin and extreme iron deficiency when systemic hepcidin concentrations are very low [47].

Macrophage ferroportin also plays an important role in iron recycling after muscle injury [48]. Selective loss of macrophage ferroportin in mice with muscle injury limited local iron recycling, impaired muscle healing and resulted in smaller myofibers and fat accumulation. 

In a mouse model of colitis, the hepcidin-ferroportin interaction in the intestine was shown to be important for mucosal healing [49]. Absence of local hepcidin production or insensitivity of macrophage ferroportin to hepcidin is thought to result in higher local extracellular iron concentrations, affecting the growth of luminal and tissue-infiltrating bacteria and worsening DSS-induced colitis. 

During pregnancy, ferroportin is very highly expressed on the basal surface of syncytiotrophoblast in the placenta [7,50], where it plays an indispensable role in iron transfer to the fetus. Placental ferroportin appears to be strongly regulated by the trophoblast intracellular iron concentrations, via the IRE-IRP system [50]. Interestingly, in normal pregnancy, fetal hepcidin concentrations are very low in mice [50] and do not appear to contribute to baseline iron homeostasis or affect iron transport through placental ferroportin. However, fetal hepatic production of hepcidin may be sufficient to act on fetal hepatic ferroportin in an autocrine/paracrine manner to retain iron in the fetal liver [51] where it is used for fetal erythropoiesis until erythropoiesis transitions to the marrow, close to birth.

## 10. Functions of the Hepcidin-Ferroportin Axis in Host Defense

Infection and inflammation induce hepcidin, predominantly through the effects of the cytokine interleukin-6 (IL-6) [52], and increased concentrations of hepcidin then inhibit ferroportin activity, leading to depletion of iron in plasma and extracellular fluid (hypoferremia). Independently of hepcidin, microbial molecules that stimulate toll-like receptors suppress cellular ferroportin mRNA transcription [34] sufficiently to cause hypoferremia but it is not clear how much this effect contributes to hypoferremia generated during infections in vivo. As ferroportin is much older on the evolutionary timeline than hepcidin, studies of the hypoferremic effects of infection and inflammation in invertebrates, which lack hepcidin, should be illuminating. 

Hypoferremia has long been suspected of having a host defense function but direct evidence for this is quite recent [53]. Since transferrin-bound iron is poorly accessible to most microbes, the most important iron species that supports microbial growth is non-transferrin bound iron (NTBI) [54]. During infections, increased cytokine concentrations inhibit erythropoiesis and activate the production of myeloid cells important for host defense [55]. The inhibition of erythropoiesis decreases the consumption of plasma iron, just as increased recycling of damaged erythrocytes and other cells during infections increases iron delivery to plasma. The imbalance would raise the saturation of transferrin by iron and enhance the production of NTBI, thereby increasing the risk of microbial outgrowth. However, inflammatory increase of hepcidin and the effects of inflammation on ferroportin transcription prevent the generation of NTBI by sequestering iron, predominantly inside macrophages. This form of “nutritional immunity” is particularly important in infections with gram negative bacteria and some fungi ([54,56,57,58]. Hepcidin can also kill microbes on contact in vitro [59,60] but the concentrations required for this direct microbicidal activity exceeded its concentrations in extracellular fluid, even in inflamed models. 

## 11. Disorders of the Hepcidin-Ferroportin System

The hepcidin-ferroportin system, in analogy to other endocrine systems, can be disrupted by mutations that affect the quantity or functionality of the ligand hepcidin or its receptor ferroportin. Loss-of-function mutations in positive regulators of hepcidin or the hepcidin gene itself (Table 2) cause hereditary hemochromatosis [61], a set of diseases characterized by increased absorption of dietary iron, often causing massively increased body iron content, and high plasma iron concentrations that saturate the iron-binding capacity of transferrin, leading to the appearance of non-transferrin-bound iron (NTBI). In affected patients who are not treated, NTBI is taken up avidly through alternative iron transporters [62] in the liver, pancreas, heart and endocrine glands, causing progressive tissue injury, organ failure, and even premature death. Two forms of hemochromatosis define its clinical spectrum. At one extreme is the severe juvenile form that can cause severe heart disease, endocrinopathy and death in young adults and at the other extreme are much less severe adult forms which can even be clinically silent and not cause organ damage. Loss-of-function ferroportin mutations cause iron overload that is usually localized to macrophages, cells that use ferroportin to export iron recycled from senescent erythrocytes, and causes increased serum ferritin but not clinically significant disease.

Loss-of-function mutations in the negative regulator of hepcidin transcription, the transmembrane serine protease TMPRSS6 (also called matriptase 2), in mice or humans cause a disease “iron-refractory iron deficiency anemia” (IRIDA) where hepcidin is excessive despite severe iron deficiency [63,64]. Elevated hepcidin inhibits intestinal iron absorption and the release of stored and recycled iron, leading to low plasma iron concentrations and a microcytic anemia.

Hepcidin concentrations can also become dysregulated as a consequence of diseases that affect iron absorption from the diet or cause a loss of blood from the body (each 1 mL of packed erythrocytes represents about 1 mg of iron), produce systemic inflammation, make erythropoiesis ineffective (lots of erythrocyte precursors but few mature) or cause anemia requiring erythrocyte transfusions without matching blood loss. These disease pathways are summarized in Table 3. Iron deficiency, iron deficiency anemia [65] and anemia of inflammation [66] are very common disorders affecting a large portion of the population. 

## 12. Summary

The hepcidin-ferroportin system evolved to maintain stable body iron content and plasma iron concentrations. The system also plays an important role in innate immunity, particularly in host defense against gram-negative bacteria and some fungi. Dysregulation of hepcidin or ferroportin production or their interaction underlies the pathogenesis of a spectrum of iron disorders, from iron restrictive anemias to iron overload conditions. Thus, plasma hepcidin measurements may be useful for diagnosing iron disorders, and therapeutic targeting of the hepcidin-ferroportin system is a promising new direction to develop improved treatments for iron disorders. 

## Figures and Tables

**Table 1:** Hepcidin-independent mechanisms that regulate ferroportin.

Mechanism	Mode	Cell Type	Cellular Effect on FPN	Note
IRE-IRP [35]	translational	macrophage	↑ cellular iron = ↑ FPN	This mechanism is bypassed by ferroportin transcripts lacking the 5′IRE, in erythroid cells and duodenal enterocytes
Iron via Nrf2 [36]	transcriptional	macrophage	↑ cellular iron = ↑ FPN	Requires high iron concentrations
Heme via BACH and Nrf2 [36]	transcriptional	macrophage	↑ cellular heme = ↑ FPN	Induces ferroportin in macrophages that recycle iron from hemoglobin and heme
miR-485–3p [37]	translational	multiple cell types	↓ cellular iron = ↓ FPN	Physiological role is uncertain
TLR [34]	transcriptional	macrophage	Ligands ↓ FPN	Unclear how much this mechanism contributes to responses to infections
HIF2α [38]	transcriptional	enterocyte	↓ cellular iron = ↑ FPN	May induce ferroportin mRNA in enterocytes during low hepcidin states
miR-20a and miR-20b [39,40]	translational	enterocyte, lung	↑ miRNA = ↓ FPN	Physiological role is uncertain

**Table 2:** Forms of hereditary hemochromatosis.

Mutated Gene	Function	Hepcidin	Tissue Iron	Clinical Severity
HFE	Loss	Low	Liver, heart, endocrine	Moderate, adult form
TFR2	Loss	Low	Liver, heart, endocrine	Moderate, adult form
Hemojuvelin	Loss	Very low	Heart, endocrine, liver	Severe, juvenile form
Hepcidin	Loss	Very low to absent	Heart, endocrine, liver	Severe, juvenile form
Ferroportin	Gain	High	Liver, heart, endocrine	Variable, some severe
Ferroportin	Loss	Normal or low	Spleen macrophages	Mild

**Table 3:** Disorders with dysregulated hepcidin production.

Disease State	Serum Hepcidin	Plasma Iron	Body Iron	Common Phenotypes
Iron deficiency	Low	Low	Low	Fatigue, anemia
Systemic inflammation	High	Low	Normal	Fatigue, anemia
Ineffective erythropoiesis	Low	High	Increased	Like hemochromatosis
Erythrocyte transfusions	High	High	Increased	Like hemochromatosis

## Data Availability

Not applicable.
